# Spinal Mobility Limitation Can Be the Main Reason of Kinesiophobia in Ankylosing Spondylitis

**DOI:** 10.7759/cureus.42528

**Published:** 2023-07-27

**Authors:** Ilker Fatih Sari, Samet Tatli, Ilker Ilhanli, Evren Er, Zerrin Kasap, Nurçe Çilesizoğlu Yavuz, Fazil Kulakli

**Affiliations:** 1 Physical Medicine and Rehabilitation, Giresun University Faculty of Medicine, Giresun, TUR; 2 Physical Medicine and Rehabilitation, Bingöl State Hospital, Bingöl, TUR; 3 Physical Medicine and Rehabilitation, Ondokuz Mayis University Faculty of Medicine, Samsun, TUR; 4 Physical Medicine and Rehabilitation, Erbaa State Hospital, Tokat, TUR

**Keywords:** disease activity, functional status, quality of life, spinal mobility, ankylosing spondylitis, kinesiophobia

## Abstract

Objective

The aim of this study is to determine the presence of kinesiophobia in patients with ankylosing spondylitis (AS) and to examine the factors affecting kinesiophobia.

Materials and methods

Sixty patients with AS participated in the study. Kinesiophobia was evaluated using the Tampa Scale for Kinesiophobia (TSK). Disease activity was assessed using the Bath AS Disease Activity Index (BASDAI) and AS Disease Activity Score with C-reactive protein (ASDAS-CRP), functional status using the Bath AS Functional Index (BASFI), spinal mobility using the Bath AS Metrology Index (BASMI), and quality of life using the AS Quality of Life Questionnaire (ASQoL). Those with a TSK score of >37 were classified as patients with high kinesiophobia, while those with a score of ≤37 as patients with low kinesiophobia.

Results

High kinesiophobia was detected in 29 (48.3%) patients. Age, disease duration, BASDAI, ASDAS-CRP, BASFI, ASQoL, and BASMI values were higher in these patients. The TSK scores correlated with age, duration of disease, ASDAS-CRP, BASFI, BASMI, and ASQoL (r = 0.697, r = 0.600, r = 0.410, r = 0.690, r = 0.889, and r = 0.576, respectively). As a result of the multivariate binary logistic regression analysis, BASMI was found to be the only statistically significant factor for high kinesiophobia (OR 5.338, 95% CI: 1.133-25.159, p = 0.034).

Conclusion

Kinesiophobia is seen at a high rate in patients with AS. In this study, the most important risk factor for kinesiophobia is found to be decreased spinal mobility. To prevent kinesiophobia - which prevents exercise, the cornerstone of AS treatment - patients should be encouraged to exercise and be active.

## Introduction

Ankylosing spondylitis (AS) is one of the most common inflammatory diseases that affect the axial skeleton. It is characterized by inflammatory back pain, causes structural and functional limitations, and reduces the quality of life (QoL) [1]. The aim of AS treatment is to control symptoms and inflammation, prevent progressive structural damage, and maximize QoL [2,3]. AS can be managed through a combination of pharmacological and non-pharmacological interventions. Non-pharmacological treatment plays an important role in optimal AS treatment [2,3]. Exercise and physiotherapy hold an important place among the non-pharmacological treatment recommendations of the Assessment of Spondyloarthritis International Society (ASAS)-European Alliance of Associations for Rheumatology (EULAR) and American College of Rheumatology (ACR) in axial spondyloarthritis management [2,3]. Unrelated to pharmacological treatment (nonsteroidal anti-inflammatory drugs, sulfasalazine, tumour necrosis factor inhibitors, interleukin-17 inhibitors, Janus kinase inhibitors), there is substantial evidence that exercise has beneficial effects on AS outcomes [2].

Kinesiophobia is the fear of movement with the thought that it may cause injury or re-injury [4]. It was first described by Kori et al. [5]*.* Physical activity and exercise are important in the management of chronic musculoskeletal diseases and have positive effects on reducing pain and disability [6,7]. However, some patients experience fear of moving into chronic musculoskeletal diseases. This prevents exercise, which is an important part of treatment. As a result of not being able to exercise, the negative effects of the disease increase [7].

As mentioned before, AS is a chronic inflammatory disease with pain, and exercise plays an important role in AS management. This suggests that conditions such as pain [8], structural changes [9], and deterioration in the quality of life [8] in patients with AS can cause kinesiophobia. A few studies have investigated the factors (age, disease duration, disease activity, quality of life, spinal mobility and physical function limitation, etc.) associated with the presence of kinesiophobia in AS, but the design of these studies did not include regression analysis. For this reason, these studies did not report any independent factors [8,9]. Based on this idea, this study aims to determine the presence of kinesiophobia in patients with AS, the factors that affect kinesiophobia in these patients, and the effect ratios of these factors.

## Materials and methods

The study protocol and design were approved by the Clinical Research Ethics Committee of Giresun Training and Research Hospital (decision date: 16.01.2023, number KAEK-003). All participants provided written informed consent, and the study was performed in accordance with the Declaration of Helsinki.

This is a prospective cross-sectional study conducted at the Giresun University Giresun Training and Research Hospital Physical Therapy and Rehabilitation Outpatient Clinic between January 2023 and May 2023. Patients between the ages of 18 and 65 years diagnosed with AS according to the modified New York Criteria and meeting the ASAS classification criteria were included in the study [10,11].

Patients with a history of surgical procedures related to the musculoskeletal system in the last 12 months, who had taken intra-articular or ligament injections within the last 1 month, had difficulty in movement due to neurological and/or vestibular diseases, had an active infection that could affect acute phase reactants, or were illiterate or unable to fill in the questionnaires were excluded from the study.

Assessment

The study participants were asked about their demographic characteristics, smoking status, and comorbid diseases. Duration of AS (years), acute phase reactants [recommended C-reactive protein (CRP) (mg/dl)], and human leukocyte antigen (HLA)-B27 positivity were recorded. In addition, their disease activity, functional capacity, spinal mobility, quality of life, and kinesiophobia were evaluated.

Disease activity

The Bath Ankylosing Spondylitis Disease Activity Index (BASDAI) and Ankylosing Spondylitis Disease Activity Score with C-reactive protein (ASDAS-CRP) were used to determine the disease activity.

The BASDAI is a scale that determines patient-reported disease activity. It comprises six questions, which evaluate five main symptoms (Q1, fatigue; Q2, spinal pain; Q3, peripheral joint pain or swelling; Q4, localized tender areas; Q5, severity of morning stiffness; Q6, duration of morning stiffness). Each question was evaluated with a 0-10 cm visual analog scale. The duration of morning stiffness was determined by dividing 0-2 hours at intervals of 1/2 hour on a 10 cm chart. The final score obtained by evaluating the scores obtained on the six questions ranges between 0 and 10; higher values indicate higher disease activity. The BASDAI formula: [(Q1 + Q2 +Q3 + Q4) + (Q5 + Q6)/2]/5 [12].

The ASDAS‐CRP is a measurement of disease activity comprising patient-reported components (Q2, spinal pain; Q3, peripheral joint pain or swelling, and Q6, duration of morning stiffness) from the BASDAI, patient global assessment of disease activity (PGA), and CRP (mg/L). Higher values indicate higher disease activity. ASDAS-CRP was calculated using the following formula: 0.12XQ2 + 0.06XQ6 + 0.11XPGA + 0.07XQ3 + 0.58XLn (CRP+1) [12,13].

Physical functional level

The Bath Ankylosing Spondylitis Functional Index (BASFI) was used to evaluate the functional capacity of the patients. This scale comprises 10 items, which question the functionality of the patient in daily living activities. Higher values indicate more dysfunction [14].

Spinal mobility and chest expansion

The spinal mobility of the patients was evaluated using the Bath Ankylosing Spondylitis Metrology Index (BASMI). Tragus-wall distance (cm), modified Schober’s test (cm), thoracolumbar lateral flexion (cm), cervical rotation (degree), and intermalleolar distance (cm) are evaluated within the BASMI, where high values indicate a decrease in spinal mobility [14]. In addition, the chest expansion of the patients was also measured.

Quality of life

The quality of life of the patients with AS was evaluated using the Turkish version of the Ankylosing Spondylitis Quality of Life Questionnaire (ASQoL). The ASQoL measures the quality of life of patients from the yes-no answers provided by them for each question over 18 items [15]. High scores indicate impaired quality of life [15].

Assessment of kinesiophobia

Kinesiophobia was evaluated using the Tampa Scale for Kinesiophobia (TSK), developed by Kori et al. [5]. It comprises 17 items, each of which is evaluated with a 4-point Likert score. The total score varies between 17 and 68; higher scores indicate higher kinesiophobia [5]. In this study, the patients who scored above 37 were classified into the “high kinesiophobia” group, and those who scored 37 and below were classified into the “low kinesiophobia” group [16].

Sample size

G*Power (V3.1) software (Informer Technologies, Inc., Los Angeles, USA) was used to calculate the required sample size. Using data from a previous study, the effect size in our sample size calculation was found to be 0.76 [8]. Based on a power of 80% and a 5% level of significance, the total sample size required was calculated as 58.

Statistical analyses 

Statistical analysis was performed using SPSS version 23.0 (IBM Corporation, Armonk, USA). Continuous variables were expressed as mean ± standard deviation (SD) and median (minimum-maximum), and categorical variables were reported as numbers and frequencies. Normality was assessed using the Shapiro-Wilk test. Quantitative data between the groups were compared using the independent samples t-test or Mann-Whitney U test according to the normality of the data. Pearson’s chi-square or Fisher’s exact test was used to compare categorical data between the groups. The Spearman correlation coefficient was used to evaluate the relationships among the quantitative variables. Risk factors affecting high kinesiophobia were examined using binary logistic regression analysis and the Enter method. Additionally, for the relationship between TSK and other variables, a linear regression analysis was performed and a scatter plot was created. Statistical significance was accepted as p < 0.05.

## Results

A total of 60 patients with AS, 42 male and 18 female, were included in the study. The mean age of the patients was 40.85 ± 10.72 years. High kinesiophobia was present in 29 (48.3%) patients, and low kinesiophobia was present in 31 (51.7%) patients.

Table 1 compares the demographic data and comorbid conditions of patients with low and high kinesiophobia. The mean age was higher in those with high kinesiophobia (p < 0.001). There was no difference between the two groups in terms of gender, smoking status, comorbid hypertension, and diabetes mellitus. HLA-B27 was positive in 20 (64.5%) patients with low kinesiophobia and in 22 (72.5%) patients with high kinesiophobia, and there was no statistical difference between the two groups (p = 0.338). Twenty-one (67.7%) patients with low kinesiophobia and 24 (82.8%) patients with high kinesiophobia were found to be using biological disease-modifying antirheumatic drugs (DMARDs). There was no statistical difference between the two groups in terms of biological DMARDs use (p = 0.179).

**Table 1:** Comparison of some demographic and comorbid conditions of ankylosing spondylitis patients with low and high kinesiophobia ^t ^Independent Sample T-Test; ^p ^Pearson Chi-Square;^ f^ Fisher’s Exact Test;  mean ± standard deviation; number (%)

	Low Kinesiophobia (n=31)	High Kinesiophobia (n=29)	p value
Age (years)	34.77±9.42	47.35 ± 7.89	<0.001^t^
Gender			0.866^p^
Female	9 (29.0)	9 (31.0)	
Male	22 (71.0)	20 (69.0)	
Smoking	8 (25.8)	12 (41.4)	0.201^p^
Hypertension	7 (22.6)	10 (34.5)	0.307^p^
Diabetes mellitus	2 (6.5)	5 (17.2)	0.247^f^

The AS-related parameters of patients with low and high kinesiophobia are shown in Table 2. Disease duration was longer in patients with high kinesiophobia (p < 0.001). Disease activity (BASDAI and ASDAS-CRP) was higher in patients with high kinesiophobia (p < 0.001 and p = 0.001, respectively). AS patients with high kinesiophobia had greater impairment in functional capacity (BASFI), quality of life (ASQoL), and spinal mobility (BASMI) (all p < 0.001). In all physical examination measurements (Tragus-wall distance, modified Schober’s test, thoracolumbar lateral flexion, cervical rotation, intermalleolar distance, and chest expansion), limitations were more common in those with high kinesiophobia (all p < 0.001).

**Table 2:** Comparison of disease activity scales, quality of life and physical examination findings of ankylosing spondylitis patients with low and high kinesiophobia BASDAI: Bath Ankylosing Spondylitis Disease Activity Index; ASDAS: Ankylosing Spondylitis Disease Activity Score; CRP: C-reactive protein; BASFI: Bath Ankylosing Spondylitis Functional Index; BASMI: Bath Ankylosing Spondylitis Metrology Index; ASQoL: Ankylosing Spondylitis Quality of Life, ^m^ Mann-Whitney U test; ^t^ Independent Sample T-Test; mean ± standard deviation; median (minimum-maximum)

	Tampa Scale for Kinesiophobia Scores				p value
	Low Kinesiophobia		High Kinesiophobia		
Disease duration, (years)	9.32 ± 7.45	6 (1 - 27)	18.1 ± 9.32	19 (4 - 36)	<0.001^m^
BASDAI	4.43±1.74	4.2 (1.4 - 7.5)	6.63 ± 1.88	7 (1.6 - 9.10)	<0.001^m^
ASDAS-CRP	2.85 ± 0.95	2.8 (0.9 - 4.6)	3.68 ± 0.88	3.9 (1.5 - 5)	0.001^t^
BASFI	2.87 ± 2.13	2.7 (0.2 - 8.2)	6.29 ± 2.15	6.5 (0.7 - 9.4)	<0.001^m^
BASMI					
Lateral lumbar flexion (cm)	15.92 ± 4.36	15.5 (8 - 26)	8.01 ± 3.95	7.5 (2.4 - 17)	<0.001^t^
Tragus to Wall distance (cm)	12.29 ± 2.06	12 (8 - 18)	19.7 ± 7.98	16 (9.5 - 38.5)	<0.001^m^
Modified Schober’s (cm)	5.21 ± 1.32	5.5 (3 - 8.5)	2.51 ± 1.61	2 (0.2 - 5.5)	<0.001^t^
Intermalleolar Distance (cm)	99.19 ± 16.88	99 (58 - 130)	82.9 ± 16.15	84 (50 - 110)	<0.001^t^
Cervical Rotation (degree)	80.23 ± 7.23	82 (60 - 90)	54.31 ± 24.34	61 (7 - 83)	<0.001^m^
Total	2.16 ± 0.85	2 (0.8 - 4)	5.08 ± 1.82	4.8 (1.6 - 8.2)	<0.001^t^
Chest Expansion (cm)	5.91 ± 1.47	6 (3 - 8.5)	3.12 ± 1.36	3 (1.3 - 6.5)	<0.001^t^
ASQoL	8.19 ± 4.37	8 (0 - 16)	13.55 ± 4.54	15 (1 - 18)	<0.001^m^

The relationship between the TSK scores and disease-related parameters is shown in Table 3. Kinesiophobia was positively correlated with BASMI, age, disease duration, BASFI, ASDAS-CRP, and ASQoL (r = 0.889, r = 0.697, r = 0.600, r = 0.690, r = 0.410, and r = 0.576, respectively). The results of physical examination indicated a negative correlation between TSK scores and modified Schober’s test, thoracolumbar lateral flexion, cervical rotation, chest expansion, and intermalleolar distance, and a positive correlation with the Tragus-wall distance (r = -0.818, r = -0.791, r = -0.797, r = -0.776, r = -0.561, and r = 0.673, respectively).

**Table 3:** Correlation analysis of Tampa scale for kinesiophobia scores TSK: Tampa Scale for Kinesiophobia;  ASDAS: Ankylosing Spondylitis Disease Activity Score; CRP: C-reactive protein;  BASFI: Bath Ankylosing Spondylitis Functional Index; BASMI: Bath Ankylosing Spondylitis Metrology Index; ASQoL: Ankylosing Spondylitis Quality of Life; r: Spearman's Rho correlation coefficient

	TSK (r)	P value
Age, (years)	0.697	<0.001
Disease duration, (years)	0.600	<0.001
ASDAS CRP	0.410	<0.001
BASFI	0.690	<0.001
ASQoL	0.576	<0.001
BASMI		
Lateral lumbar flexion	-0.791	<0.001
Tragus to Wall distance	0.673	<0.001
Modified Schober’s Test	-0.818	<0.001
Intermalleolar Distance	-0.561	<0.001
Cervical Rotation	-0.797	<0.001
Total	0.889	<0.001
Chest Expansion	-0.776	<0.001

**Figure 1:**
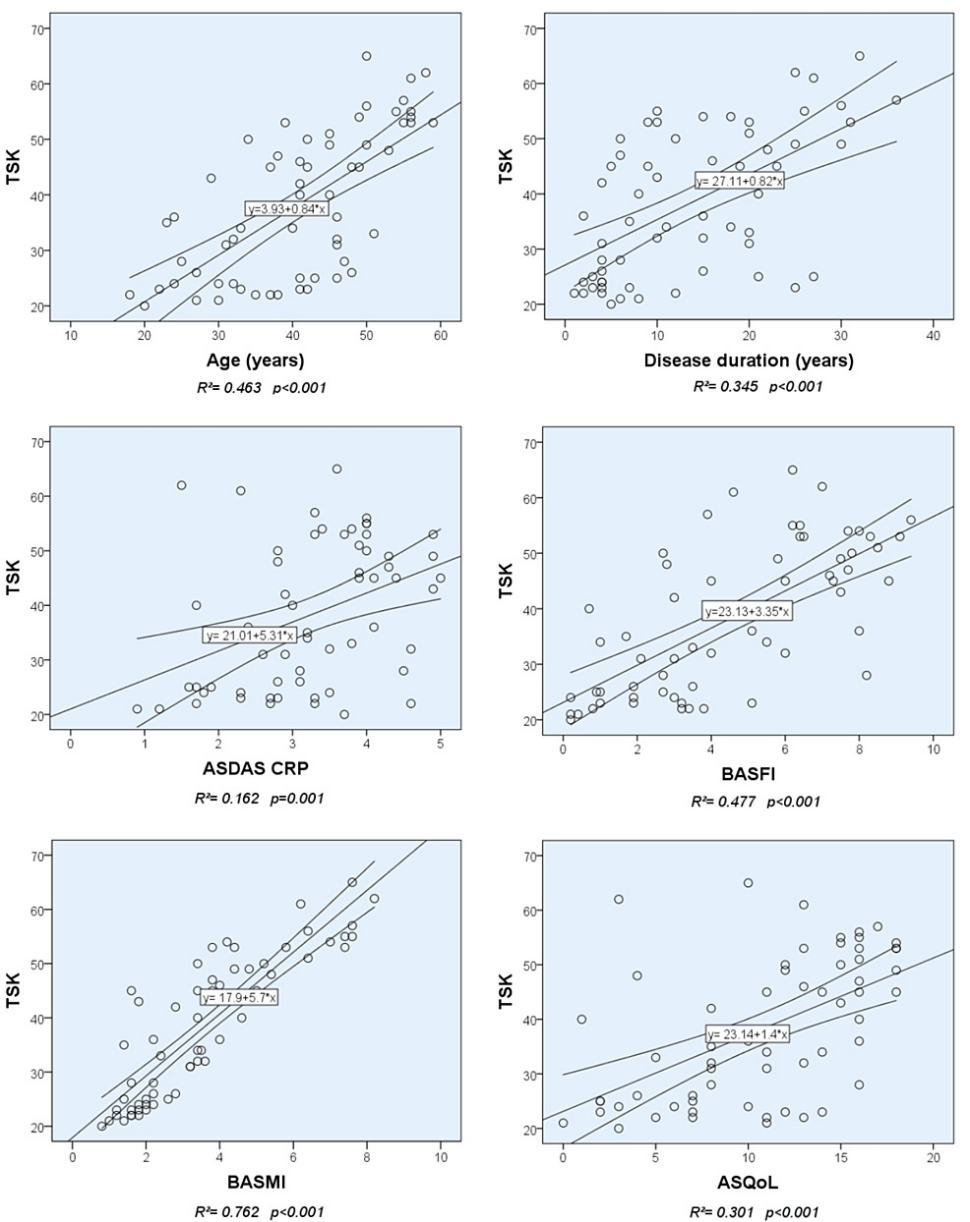
Relationship between TSK scores and variables tested (ages, disease duration, ASDAS CRP, BASFI, BASMI and ASQoL) with a scatter plot. TSK: Tampa Scale for Kinesiophobia;  ASDAS: Ankylosing Spondylitis Disease Activity Score; CRP: C reactive protein;  BASFI: Bath Ankylosing Spondylitis Functional Index; BASMI: Bath Ankylosing Spondylitis Metrology Index; ASQoL: Ankylosing Spondylitis Quality of Life

In addition to the correlation analysis, a linear regression analysis was performed to examine a possible relationship between TSK scores and age, disease duration, ASDAS CRP, BASFI, BASMI, and ASQoL. Figure 1 shows a scatter plot of the TSK scores on age, disease duration, ASDAS CRP, BASFI, BASMI, and ASQoL with regression line and 95% CI. There was a statistically significant relationship between the TSK scores and age (R^2^ = 0.463; p < 0.001), disease duration (R^2 ^= 0.345; p < 0.001), ASDAS-CRP (R^2^ = 0.152; p = 0.001), BASFI (R^2^ = 0.477, p < 0.001), BASMI (R^2^ = 0.762, p < 0.001), and ASQoL (R^2^ = 0.301, p < 0.001).

Risk factors affecting high kinesiophobia were analyzed using binary logistic regression analysis with the Enter method (Table 4). When the model was examined as univariate, the increase in age, disease duration, ASDAS CRP, BASMI, and ASQoL values increased the risk of high kinesiophobia (p < 0.001, p = 0.001, p = 0.003, p < 0.001, p < 0.001, and p < 0.001, respectively). When the model was examined as multivariate, only BASMI significantly increased the risk of developing high kinesiophobia (OR:5.338; p = 0.034).

**Table 4:** Evaluation of factors affecting high kinesiophobia by Binary Logistic Regression analysis ASDAS: Ankylosing Spondylitis Disease Activity Score; CRP: C-reactive protein;  BASFI: Bath Ankylosing Spondylitis Functional Index; BASMI: Bath Ankylosing Spondylitis Metrology Index; ASQoL: Ankylosing Spondylitis Quality of Life

	Univariate		Multivariate	
	OR (%95 CI)	p value	OR (%95 CI)	p value
Age, (years)	1.174 (1.081 - 1.274)	<0.001	1.174 (0.997 – 1.383)	0.054
Disease duration, (years)	1.126 (1.05 - 1.208)	0.001	0.894 (0.725 – 1.102)	0.292
ASDAS CRP	2.658 (1.39 - 5.081)	0.003	3.076 (0.399 – 23.706)	0.281
BASFI	1.897 (1.401 - 2.569)	<0.001	1.181 (0.485 – 2.877)	0.714
ASQoL	1.295 (1.125 - 1.491)	<0.001	1.079 (0.737 – 1.578)	0.697
BASMI	5.545 (2.318 - 13.267)	<0.001	5.338 (1.133 – 25.159)	0.034
Cox & Snell R^2^ = %61.2; Nagelkerke R^2^= %81.7				

## Discussion

There are studies in the literature which were conducted on kinesiophobia in AS; however, to the best of our knowledge, this is the first study to investigate the factors contributing to high kinesiophobia in AS by using regression analysis with modeling [8,9]. In addition, this is the first study to evaluate the ASDAS-CRP, which is the most valid, reliable, and sensitive-to-change measure of diseases, combining patient-reported outcomes and acute phase reactants (recommended CRP) [17]. The results of this study indicated that AS patients with high kinesiophobia were older, had longer disease duration, had higher disease activity, had lower functional capacity and spinal mobility, and had lower quality of life. In addition, the most important parameter increasing the risk of high kinesiophobia was an increase in the BASMI score, which indicates a decrease in spinal mobility.

AS is a chronic inflammatory disease, and patients with AS complain of chronic pain. AS causes both pain and structural changes by affecting the peripheral joints, especially the axial skeleton system [1]. These structural changes and pain impair the functional capacity and quality of life of patients [18]. In the nature of inflammatory low back pain, which is a common symptom of this disease, the pain decreases with movement and increases with rest [19]. In the treatment of patients with AS, exercise and movement are indispensable parts of the treatment [20]. However, it has been shown that the rate of patients with high kinesiophobia is high in patients with AS [8]. We consider that the presence of kinesiophobia may adversely affect the disease prognosis due to the fear of moving.

The highest TSK score was found in low back pain in patients with chronic musculoskeletal pain [21]. Chronic inflammatory low back pain is one of the most common symptoms in patients with AS. Er and AngIn [9] found the TSK score to be 41.65 ± 7.59 in patients with AS, and Oskay et al. [8] found high kinesiophobia in 66.6% of AS patients. In our study, while the mean TSK score of the patients was 38.28 ± 13.42, high kinesiophobia was detected in 48.3% of the patients. In this study, the rate of high kinesiophobia and the mean TSK score were found to be slightly lower than in those studies [8,9].

In our study, it was shown that the TSK scores increased in AS patients with age and disease duration. There may be an increase in kinesiophobia because more structural changes will develop as the disease becomes chronic, and as age increases, there may be an increase in pain not only due to inflammatory but also mechanical reasons [22]. This situation suggests that kinesiophobia can increase due to increasing structural deformities and age-related osteoarthritic changes with the chronicity of the disease [23,24].

BASDAI is one of the most valid parameters in the measurement of disease activities in AS. In previous studies on kinesiophobia, no statistically significant relationship was found between BASDAI scores and kinesiophobia [8,9]. In our study, unlike in other studies, BASDAI scores were found to be higher in those with high kinesiophobia. In addition, a positive correlation was found between the disease activity measured using the BASDAI and TSK scores. Mean BASDAI scores were 4.34±1.91 in one of these studies [9] and 1.06 ± 0.75 in the low kinesiophobia group, 1.34 ± 0.96 in the high kinesiophobia group in the other study [8]. However, in our study, the mean BASDAI score was 4.43±1.74 in low kinesiophobia, 6.63 ± 1.88 in high kinesiophobia, and it was way beyond these studies. Regarding BASDAI <4 refers to remission, patients in these studies were mostly in remission. We considered that for this reason, we found a correlation between kinesiophobia and BASDAI. In addition to the traditional BASDAI used in the evaluation of disease activity in recent years, the ASDAS-CRP, which is more recommended today, is another scale that has been validated and is easy to apply in clinical practice [25]. In this study, a moderately positive correlation was found between ASDAS-CRP and kinesiophobia. Increased disease activity severity, increase in pain, decrease in functionality, and movement capacities of individuals will cause the development of kinesiophobia.

AS is known to cause functional disabilities. Patients with AS have difficulty performing certain daily life functions [26]. In this study, an increase in the development of kinesiophobia was observed with a decrease in functionality. While BASFI scores were higher in those with high kinesiophobia, a high degree of positive correlation was found between the TSK and BASFI scores. Oskay et al. and Er and AngIn found a correlation between the BASFI and TSK scores [8,9]. In Oskay et al.’s [8] study, BASFI scores were also found to be higher in patients with high kinesiophobia. Conditions such as active disease and decreased spinal mobility can reduce the functionality of patients. However, it is not easy to determine the cause-effect relationship between kinesiophobia and functional disability. Decreased functionality increases kinesiophobia, and increased kinesiophobia can further reduce the functionality of patients. Thus, we consider that both situations affect each other.

AS affects the QoL of patients negatively in both daily and work life due to reasons such as pain, structural changes, and the functional insufficiency that it may cause [27]. In this study, a moderate correlation was found between TSK and ASQoL scores. The patients with high kinesiophobia had higher ASQoL scores. Oskay et al. [8] also found similar results in their own study. The fact that patients experience fear of moving might make them less active, have decreased functionality, and be unable to perform daily living activities. These conditions will adversely affect QoL.

The most crucial point of this study is that when a multivariate regression analysis is performed, the decrease in spinal mobility emerges as the most significant factor and the only one that is statistically significant, increasing the development of high kinesiophobia. When analyzing other studies evaluating kinesiophobia in patients with AS, Er and AngIn [9] found a moderate correlation between the BASMI and TSK scores, while Oskay et al. [8] found no correlation. In this study, a very strong correlation was found between the scores, and the increase in the BASMI score was found to be the most contributing factor to high kinesiophobia as a result of the multivariate regression analysis. AS causes a range of motion limitations and posture disorders, especially in the spine and hips, due to inflammation and structural damage. We consider that kinesiophobia is increasingly severe in these patients due to the social isolation that may be caused by postural disorders and changes in gait patterns, as well as pain and limitation in the range of motion of the joint [28]. AS causes joint movement limitations and posture disorders, especially in the spine and hips, due to inflammation and structural damage. We consider that kinesiophobia is increasingly severe in these patients due to the social isolation that may be caused by postural disorders and changes in gait patterns, as well as pain and limitation in the joint’s range of motion [28]. For these reasons, the fear of moving due to increased kinesiophobia in patients with AS will limit their physical activity and exercise. This will cause more immobilization and prevent exercise, which is one of the most important cornerstones of AS treatment. Thus, to prevent structural changes in patients with AS, increase their functional capacity, and maximize their quality of life, patients should be relieved from kinesiophobia by making them more active through exercise.

This study has several limitations. First, because of the cross-sectional study design, a prospective longitudinal study is needed. Second, the study was single-center and patients were accepted regardless of whether the disease was remission or active. And also the markers like inflammatory back pain, morning stiffness, CRP, structural damages, and psychological factors which may affect kinesiophobia individually could not be analysed separately because of the small sample size. 

## Conclusions

Kinesiophobia is common in patients with AS and it is a factor in reduction of motion in AS. AS patients with kinesiophobia avoid moving and also exercising because of their fear. Especially reduction in spinal mobility, structural changes, and limited range of joint movement should be taken into consideration for kinesiophobia in AS. Regarding that exercise is the cornerstone of treatment in the management of AS, kinesiophobia is a handicap in the prognosis of the disease. To prevent kinesiophobia and spinal mobility deficit, patients should be encouraged to exercise and be physically active. It is thought that with further studies, including a larger sample size, besides the disease activity scores, every individual factor which may affect disease activity may be analysed for kinesiophobia in AS.
